# Comparison of Reconstructive Materials in Paediatric Orbital Fractures: A Systematic Review

**DOI:** 10.3390/cmtr19010012

**Published:** 2026-02-23

**Authors:** Jane Chen, Anton Sklavos, Mustafa Mian, Ricky Kumar

**Affiliations:** 1 Department of Oral and Maxillofacial Surgery, Monash Health, 135 David Street, Dandenong, VIC 3175, Australia; 2 Plastic and Maxillofacial Surgery Department, The Royal Children’s Hospital, 50 Flemington Road, Parkville, VIC 3052, Australia

**Keywords:** orbital fracture, paediatrics, resorbable implants, autografts, alloys, polymers, titanium, fracture fixation, reconstructive surgical procedures

## Abstract

Paediatric orbital fractures require careful reconstruction to prevent long-term functional and aesthetic sequelae. Material selection is critical due to the anatomical and developmental considerations unique to children. Comparative data to guide decision making remain sparse and inconclusive. A systematic search was conducted in PubMed, Scopus, Web of Science, and Embase (through February 2025), following Preferred Reporting Items for Systematic Review and Meta-analyses (PRISMA) guidelines. Studies reporting outcomes and/or complications associated with implant materials used in the reconstruction of paediatric orbital fractures were included. Outcomes included postoperative diplopia, enophthalmos, restriction of eye movements, removal of material, and return to theatre (RTT). In total, 54 studies encompassing a total of 562 patients and 563 implants were included. Polymers (*n* = 169), alloplasts (*n* = 167) and autologous (*n* = 166) implants were the most commonly used reconstructive material. Late postoperative diplopia occurred in 7% of polymers (12/169), 6% of alloplasts (10/167), 29% of allografts (6/21), 24% of xenografts (6/25) and 33% of metals (2/6). Reported enophthalmos was highest in the autologous group (8%) but was only reported in 34 of the 54 studies. Infection, removal of implant material and RTT were low across all groups (1–4%). No donor site morbidity was reported. Robust studies with standardised outcomes and adequate follow-up are needed to inform evidence-based material selection in paediatric orbital reconstruction.

## 1. Introduction

Orbital fractures account for 2.1% to 23% of maxillofacial fractures in the paediatric population, with the reported prevalence varying depending on age and geographical location [1,2,3,4]. These fractures are typically sustained due to motor vehicle accidents, bicycle accidents, sporting-related injury, falls and interpersonal violence. Males are disproportionately represented in the injured population [2,3,5,6,7]. Limited patient compliance to examination and radiographic investigations may pose a barrier to accurate diagnosis and management of orbital fractures in children [4]; therefore, a high index of suspicion should be maintained if there is an associated high-risk mechanism of injury. Inferior orbital fractures represent a common subtype of paediatric facial fractures [8,9], and, due to the propensity of the elastic paediatric bone to undergo “green-stick” bending, the fracture pattern is typically that of a trapdoor [10]. This can lead to the entrapment of extraocular eye muscles (EOM). Blow-out fractures, which also commonly affect the floor, can lead to the herniation and potential entrapment of orbital contents. However, they are less common in children due to anatomical and physiological differences in the paediatric facial skeleton [11]. Growth of the orbital skeleton peaks during the first one to two years of life, and, by approximately age eight, the orbit is around 85–90% of adult size [12]. Further growth at a rate of 1–2% per year continues well into late adolescence [13,14]. Gradual pneumatisation of adjacent sinuses also influences the paediatric orbital fracture patterns observed [15,16]. Urgent surgical intervention is indicated if entrapment of the extraocular eye muscles is present. In cases with functional and/or aesthetic concerns, such as patients with enophthalmos, persistent diplopia, acute vertical orbital dystopia, hypoglobus or large orbital floor fractures, operative management is preferable [4,16]. However, controversy persists regarding the precise size of defect warranting surgical intervention [4]. Ischaemic necrosis and fibrosis of entrapped tissues may result in persistent diplopia [17]. Enophthalmos or hypoglobus may result from increased bony orbital volume in poorly repaired or unrepaired defects, leading to facial deformity [16]. Therefore, timely and accurate surgical intervention is necessary to prevent long-term functional and aesthetic complications.

Reconstruction of orbital wall defects in the paediatric patient requires careful planning. Surgical approaches focus on repairing the fractured orbital walls and aim to restore orbital volume and globe support, with or without the placement of grafts or implants. Whilst prior systematic reviews have predominantly synthesised evidence regarding orbital fracture reconstruction materials in cohorts that encompass both adult and paediatric populations [18,19,20,21], systematic reviews exclusive to the paediatric population are currently limited to specific material categories only [22]. Unlike in adults, the constantly changing dimensions of the facial skeleton and orbital anatomy secondary to growth and development must be considered when selecting reconstructive materials [10,22]. Implant or grafts used for paediatric orbital floor reconstruction need to balance durability with resorbability, provide adequate structural stability over time, and exhibit favourable biocompatibility and safety profiles [21,23]. A wide range of resorbable and non-resorbable implant materials and grafts have been described in the literature. These include autologous bone or cartilage grafts, alloplasts, allografts, xenografts and polymers [6,10,22,23,24,25]. This diversity, however, presents the surgeon with a dilemma, as each option entails trade-offs related to stability, longevity, and potential for complications [4,26,27]. Furthermore, comparative data to guide decision making remains sparse.

To address this gap in knowledge, a systematic review of existing literature was undertaken with the aim of comparing and evaluating outcomes associated with different reconstructive materials used in the repair of paediatric orbital fractures.

## 2. Materials and Methods

This systematic review was conducted in accordance with the Preferred Reporting Items for Systematic Review and Meta-analyses (PRISMA) 2020 guidelines [28]. A review protocol was registered with the PROSPERO database (CRD420251107626).

### 2.1. Search Strategy

A comprehensive search was conducted in the following electronic databases: MEDLINE, EMBASE, Scopus, Web of Science and PubMed, and the searches were performed from database inception through to February 2025. The search strategy comprised a combination the following terms which encompassed the areas of interest (paediatric, trauma, orbital fracture, reconstruction material): “paediatric”, “pediatric”, “child”, “orbit* fracture”, “orbit* wall fracture”, “blowout fracture”, “trapdoor fracture”, “implant”, “reconstruction”, “repair”, “surgery”, “orbit* reconstruction”, “graft”, “autologous”, “xenograft”, “polymer”, “alloplast”, “material”, “resorbable material”, “titanium”. Database specific syntax was adapted accordingly. Additional relevant articles were identified by searching the reference lists of selected articles.

### 2.2. Eligibility Criteria

Study types included were randomised control trials (RCT), prospective or retrospective cohort studies, case–control studies, case series and case reports published in English, with full text available, which reported on outcomes and/or complications associated with implant materials used in reconstruction of paediatric orbital fractures. Outcomes of interest included early (<2 weeks) and late (>2 weeks) postoperative diplopia, enophthalmos, hypoglobus, restriction of eye movements, infection, haemorrhage, visual loss, return to theatre (RTT), persistent infraorbital paraesthesia, removal of reconstructive implant materials and where applicable, donor site morbidity. Articles reporting on adult patients, reconstruction for oncological, pathological or congenital deformities, and orbital roof fractures were excluded. Nonclinical studies, animal studies, conference abstracts, and systematic reviews were also excluded.

### 2.3. Data Selection and Collection

Search results were exported to Covidence systematic review software (Veritas Health Innovation, available at www.covidence.org, accessed on 3 March 2025), a web-based collaboration software platform that streamlines the production of systematic reviews [29]. Titles and abstracts were independently screened by two individuals (JC, AS). The full text of relevant articles was retrieved and further assessed against the inclusion and exclusion criteria. Data extracted included study characteristics (title, authors, year, geographic location, study design), patient demographics (age, sex, facial fracture subtype(s) and location), treatment (orbital reconstruction material type) and outcomes (functional, aesthetic, surgical outcomes, complications, duration of follow-up). Conflicts were resolved via discussion between reviewers. Risk of bias was assessed via the Cochrane RoB-2 [30] for RCTs and, given the expected paucity of RCTs, risk of bias was also assessed via the Newcastle–Ottawa Scale (NOS) [31] for observational studies. The Joanna Briggs Institute (JBI) Critical Appraisal Checklists were used for case reports and case series [32,33].

### 2.4. Data Synthesis and Analysis

Descriptive statistics were used to analyse study variables. A meta-analysis was not conducted for this systematic review due to the presence of substantial clinical and methodological heterogeneity, small sample sizes (limiting statistical power), a lack of standardisation in outcome definitions and reporting as well as inconsistent follow-up data. Furthermore, there was a high risk of bias across observational studies without confounder control, which would severely limit the validity of pooled effect estimates. For these reasons, a narrative synthesis was undertaken. This allowed for transparent characterisation of comparative outcomes and avoided overstating the certainty of findings. All statistical analysis was performed with Microsoft Excel (Version 2601, Microsoft Corp., Seattle, WA, USA), using the Real Statistics Resource Pack software (Release 9.5.5). Fisher’s exact testing was used for pairwise comparison. A *p* value of <0.05 considered significant.

## 3. Results

**Figure 1:**
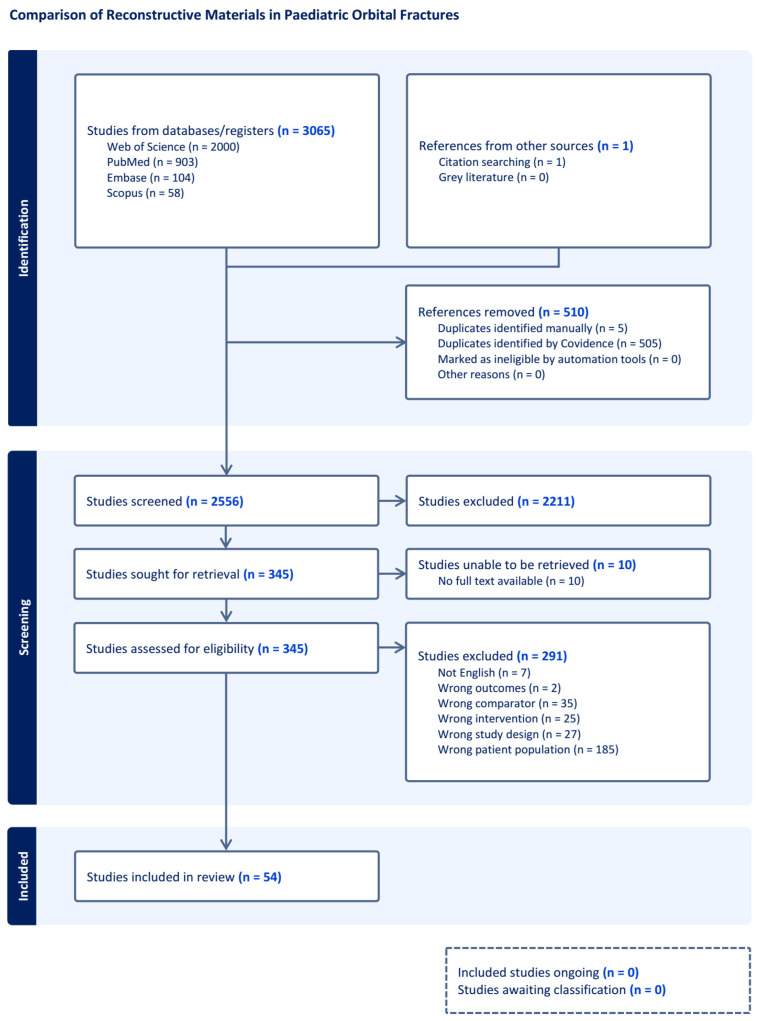
Preferred Reporting Items for Systematic Review and Meta-analyses (PRISMA) flow chart for study selection.

From 3066 studies identified in the initial search, a total of 54 studies reporting on clinical outcomes for reconstructive materials used in the repair of paediatric orbital fractures were included [6,11,34,35,36,37,38,39,40,41,42,43,44,45,46,47,48,49,50,51,52,53,54,55,56,57,58,59,60,61,62,63,64,65,66,67,68,69,70,71,72,73,74,75,76,77,78,79,80,81,82,83,84,85,86]. Figure 1 details the PRISMA protocol utilised in the review process. The study pool was comprised of 1 prospective cohort study, 30 retrospective cohort studies, 5 case series, and 18 case reports. No randomised or non-randomised control trials were identified. Studies were heterogenous and had a high risk of bias, limiting the pooling of data, as well as the ability to conduct a meaningful meta-analysis. These studies encompassed 562 unique patients and 563 reconstructive materials/implants. Three studies did not report on patient age [43,48,52]. From the studies that reported on patient age, the average age was 11 years old. Where sex was reported (46 studies), 74% (152/206) of the cohort were male. Duration of follow up was variable, ranging from 3 to 245 weeks with a mean of 52 weeks. Sample sizes were small, with an average of 10 patients and 10 implants per study. The orbital floor and/or medial walls were the most frequently fractured sites. A limited number of studies reported on concomitant zygomaticomaxillary (*n* = 6) or naso-orbito-ethmoidal fractures (*n* = 2).

The main categories of reconstructive materials utilised were polymers (*n* = 169), alloplasts (*n* = 167) and autologous grafts (*n* = 166), which constituted the majority of materials. Xenografts (*n* = 25), allografts (*n* = 21), metals (*n* = 6) and composite (*n* = 5) or combination materials (*n* = 4) were also reported but constituted much smaller sample sizes (Table 1). Both resorbable and non-resorbable implants were represented within the polymer group. Various combinations of polylactic acid (PLA), poly-L-lactide acid (PLLA), and polyglycolic acid (PGA) resorbable implants (90/166), as well as non-resorbable nylon foil (63/166), were the predominant polymer subtypes. Alloplasts were mostly represented by porous polyethylene (PPE) materials (120/167), with MedPor^®^ (Stryker, MI, USA) specifically, comprising of 34.7% of alloplastic implants. Autologous grafts mainly consisted of bone grafts with unspecified donor sites (132/166), with a minority stated to have been harvested from the calvarium (16/166) or the iliac crest (2/166). Cartilaginous grafts constituted 6% (10/166) of autologous grafts.

**Figure 2:**
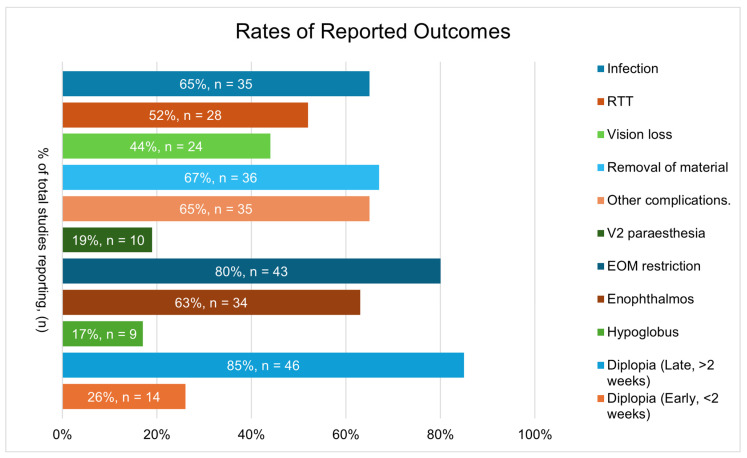
Rates of reported outcomes.

**Figure 3:**
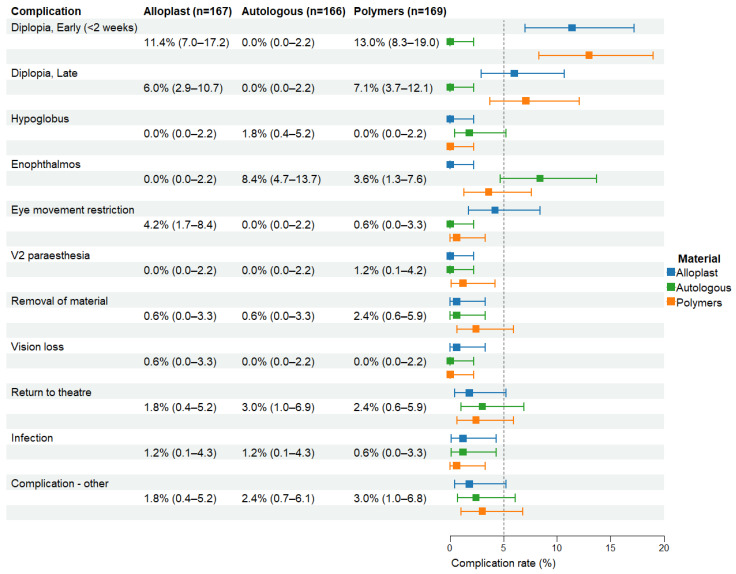
Complications by commonly used material type and clinical outcome.

Reported clinical outcomes and complication rates at last follow-up were inconsistently reported (Figure 2), with late diplopia (>2 weeks postoperatively) and EOM restriction being the most widely reported outcomes (85% and 80% respectively). The overall complication rates for each type of material and each clinical outcome are shown in Figure 3. A comprehensive table detailing complications and outcomes for all material types can be in the Supplementary Materials (Table S1). Notably, late postoperative diplopia occurred in 7% of polymers (12/169), 6% of alloplasts (10/167), 29% of allografts (6/21), 24% of xenografts (6/25) and 33% of metals (2/6). Reported enophthalmos was highest in the autologous group (8%).

Early postoperative diplopia, defined in this review as postoperative diplopia occurring within 2 weeks of surgery was highest in the polymer group (13%; 95% CI 8.3–19.0%, *n* = 22/169). This was closely followed by the alloplast group (11.4%; 95% CI 7.0–17.2%, *n* = 19/167). However, these trends were not reflected in the late postoperative diplopia incidences, defined in this study as diplopia persisting > 2 weeks post-surgery and present at last follow-up, which was observed to be highest when metal (33.3%, 95% CI 4.3–77.7%, *n* = 2/6) or allograft (28.6%, 95% CI 11.3–52.2%, *n* = 6/21) materials were used in paediatric orbital fracture reconstruction. Post-operative diplopia persisted until last follow-up (>2 weeks) in 7.1% of polymers (95% CI 3.7–12%, *n* = 12/169), 6% of alloplasts (95% CI 2.9–10.7%, *n* = 10/167) and 24% of xenografts (95% CI 9.4–45.1%, *n* = 6/25). Autogenous grafts had no reports of late diplopia (95% CI 0–2.2%). Incidence of late postoperative diplopia was statistically significantly lower in autologous grafts comparing to both alloplasts (*p* = 0.002) and polymers (*p* < 0.001). No significant difference was detected between alloplasts and polymers (*p* = 0.826). These trends were also observed at the level of subtypes, with pairwise comparison between bone grafts and PLLA, PLA and/or PGA implant materials (*p* = 0.007), as well as between porous polyethylene and bone grafts (*p* < 0.001), which was in favour of bone grafts for lower incidence of late diplopia postoperatively (Table 2).

Enophthalmos rates were observed to be highest in the autologous group at 8% (95% CI 4.7–13.7%, *n* = 14/166), compared to 3.6% (95% CI 1.3–7.6%, *n* = 6/169) in polymers and 0% (95% CI 0.0–2.2%) in alloplasts. A statistically significant difference between alloplasts and polymer groups (*p* = 0.030) as well as between the autologous and alloplast groups (*p* < 0.001) was noted. Polymers demonstrated a nonsignificant trend towards lower postoperative enophthalmos rates than the autologous grafts (*p* = 0.068).

Restriction of extraocular eye movements was observed in 4.2% (95% CI 1.7–8.4%, *n* = 7/167) of alloplasts and 0.6% (95% CI 0.0–3.3%, *n* = 1/169) of polymers and was not observed in the autologous group (95% CI 0–2.2%). Alloplasts had a higher incidence of extraocular eye movement restriction compared to autologous grafts (*p* = 0.015), but comparisons between polymers and alloplasts (*p* = 0.067) and between polymers and autologous grafts (*p* = 1) revealed no differences.

Low rates of RTT, need for implant removal, vision loss, and infection were observed across all materials (1–4%). No significant differences in overall complications were noted on pairwise comparisons between three most prevalently utilised materials. Furthermore, no donor site morbidity was recorded with regards to autologous grafts. Persistent infraorbital paraesthesia was uncommon (0–1.2%) except for the combination group (25%, 95% CI 0.6–80.6%), but this was in the context of very limited implant numbers (*n* = 4). Other uncommon but reported complications included foreign body reaction (3/563), retrobulbar haemorrhage (2/563), optic neuropathy (1/563) and a palpable graft (1/563).

### Risk of Bias

Assessment of risk of bias across the included studies revealed substantial methodological limitations, highlighting a significant risk of bias. All observational studies evaluated using the NOS were classified as poor quality, due to a lack of comparability, with a failure to control for confounders. Furthermore, the outcome reporting domain was also found to be subpar (Table S2).

Furthermore, deficiencies were present in the patient selection and outcome reporting domains. Appraisal of case series (Table S3) and case reports (Table S4) with the JBI critical appraisal checklists revealed variable reporting quality. Most case series lacking consecutive or complete patient inclusion, appropriate statistical analysis and provided insufficient detail regarding the demographic characteristics of the study population. Case reports provided sufficient information on patient demographics, clinical findings and diagnostic investigations; however, they were less comprehensive in reporting patient past medical history and detailing specifics of operative intervention or any adverse events which occurred.

In addition to substantial study heterogeneity, most included studies were deemed at significant risk of bias. The poor study quality significantly reduces the certainty of comparative findings. Therefore, the statistically significant differences demonstrated in the pairwise comparisons should be interpreted with extreme caution. These findings should not be interpreted as definite evidence of specific material superiority for their respective clinical outcomes (i.e., late postoperative diplopia, enophthalmos, EOM restriction).

## 4. Discussion

This systematic review critically appraised the existing literature regarding clinical outcomes and complications associated with reconstructive materials used in paediatric orbital fracture repair. It identified a critical knowledge gap, as evidenced by the lack of paediatric specific randomised controlled trials. Despite the high risk of bias attributable to the observational nature of studies, small sample sizes and variability of reporting on complications, the observed trends highlighted and synthesised in this review may provide relevant information when treating this under-researched population. The majority of fracture reconstructions utilised either polymers, alloplasts or autologous grafts. Amongst these, autologous grafts tended to be associated with the lowest rates of late postoperative diplopia and extraocular muscle restriction, whilst demonstrating a reasonable safety profile with no reports of donor site morbidity. In contrast, alloplastic materials were more frequently complicated by late postoperative diplopia but had a reduced incidence of postoperative enophthalmos. A range of materials was classified within polymers, and this subgroup demonstrated outcomes intermediate between autologous and alloplasts. Complications associated with reoperation, removal of implant material, significant visual loss and infection were found to be rare across all groups, highlighting the acceptable safety profiles of commonly utilised materials in contemporary paediatric orbit reconstruction.

### 4.1. Autologous Grafts

Autologous bone grafts have widely used as the “gold standard” for orbital reconstruction in paediatric craniofacial literature [4,15,21,40]. They hold both biological advantages, with an ability to integrate with the growing orbital skeleton, and mechanical advantages, such as good strength and rigidity [10,21,22,86]. These factors reduce the risk of healing complications such as infection, inflammatory reactions, graft migration, extrusion, and tethering of ocular eye muscles [4,87,88]. This corresponds with the observed findings of reduced postoperative diplopia (early and late) and freedom of eye movements. Bone grafts have been successfully sourced from the calvarium, iliac crest, rib, and the anterior maxilla [10,25,88]. However, the utilisation of autologous bone graft for orbital fracture repair presents several challenges. The finite quantity, relative inflexibility and inability to finely contour bone can affect reconstruction outcomes [25,86,89]. This can be compounded by the unpredictable rate of resorption of the graft, resulting in a loss of support and subsequent development of enophthalmos [25]. In this review, there was an 8% incidence of postoperative enophthalmos in autologous bone grafts. Furthermore, autologous bone grafts require a donor site from which the graft is harvested. This has the potential to add morbidity and operative times [10,21,22]. Notably, donor site morbidity was not observed in the current review, which may reflect a reporting bias regarding such outcomes. Autologous cartilaginous graft, namely nasoseptal and aurical cartilage, have also been reportedly used in paediatric orbital fracture repair [22,88]. However, the increased incidence of postoperative hypoglobus and enophthalmos necessitate careful case selection, as these findings may reflect a limitation of their mechanical properties, especially for large defects [22,24,27].

### 4.2. Alloplasts

Porous polyethylene (PPE) or its commercial variant, MedPor^®^, is a commonly used type of alloplastic implant in the reconstruction of paediatric orbital fractures [88]. The biocompatible material is reliable, strong and easy to contour and does not require a donor site. Its porous nature allows for fibrovascular ingrowth [89], promoting stabilisation and minimising the risk of extrusion, migration and capsule formation [22,90]. Alloplasts were observed to have lower incidence of enopthalmos when compared with autografts grafts in this review. Thus, PPE derivatives may provide adequate, long-lasting strength and stability to support the orbital tissue and restore anatomical orbital volume, especially in large defects [21,25,91]. However, despite its favourable complication profile, as documented in adult literature [21,86,87], this review finds higher rates of early and late postoperative diplopia in the alloplast group, especially within the MedPor^®^ subgroup. This may be due to extrusion, migration, fibrosis and displacement of these implants during growth which may result in injury to the neuromuscular components involved in ocular movement, thus producing diplopia [4,16,88,92]. Furthermore, alloplastic materials have been described as being associated with increased infection risk in paediatric literature [15,22]. However, the observed infection rate with PPE in this review was low (0.8%, 1/120). Alternative alloplastic materials such as polytetrafluoroethylene, share similar favourable features with PPE (biocompatibility, pliable, presence of micropores to allow for fibrovascular ingrowth), but tend to lack adequate structural strength and rigidity to support orbital content in larger defects, requiring appropriate case selection for usage [93].

### 4.3. Polymers

Resorbable polymers such as polylactic acid (PLA), poly-L-lactide acid (PLLA), polyglycolic acid (PGA), or a copolymer combination are increasingly used in paediatric orbital fracture repair [6,91]. The resorbable nature of these implants may be beneficial when balancing structural stability and support of orbital tissues within the context of a dimensionally dynamic craniofacial skeleton [22]. Resorbable materials remove the need for future operations to retrieve implanted materials as growth occurs [91]. Their disadvantages lay in their association with localised inflammatory reactions (4.3% rate), and potential lack of biocompatibility due to the residual degradation byproducts in PLLA implants [86]. Furthermore, potential functional complications such as inflammatory induced tethering of extraocular eye muscles and persistent diplopia [4,22,94] have been reported in the adult literature, with paediatric specific literature remaining sparse. These findings are consistent with those reported in this review, with two of three cases of foreign body reaction occurred in PLLA and/or PGA implants. The rate of resorption of a polymeric material influences the degree of loss of structural integrity over time and rapidly resorbing agents potentially associated with having an increased risk of enophthalmos and hypoglobus [4,25,27]. Baino et al. [25] noted that, whilst PGA had minimal inflammatory reactions, it lost structural integrity after two months and was completely resorbed by nine months. A similar finding was noted for PLAs by Wu et al. [27]. Consistent with these observations, enophthalmos cases in this review within the polymer subgroup exclusively occurred in patients repaired with PLLA, PLA +/− PGA materials. A 38% (16/90) rate of overall complications was observed in this current study and 1 of 90 PLLA, PLA +/− PGA implants were removed due to complications. Nylon foil is a non-resorbable polymeric alternative with a suitable degree of flexibility, rigidity and reduced risk of scarring or adhesion development due to its nonporous nature [27,92]; however, it consequently lacks fibrovascular ingrowth, which may contribute to increased rates of migration and extrusion [86]. Analysis of the polymer subgroup reveals variable complication rates, likely attributable to differences in resorption kinetics among the various resorbable polymer types and combinations [21]. Non-resorbable polymers represent a distinct category with their own specific complication profile.

### 4.4. Other Materials

Other materials such as allografts, xenografts, composites, and metallic and combination implants were poorly represented in the literature and findings should be interpreted with caution. Allografts such as lyophilised dura mater and xenografts such as bovine collagen derivatives (Lyoplant^®^ (B. Braun, Melsungen, Germany), Permacol™ (Medtronic, Paris, France)) are materials that may offer osteoinductive and/or osteoconductive properties [27]. However, they are less commonly used due to concerns regarding potential risk of disease transmission [21,25]. No studies considered in the review specifically reported on this type of complication. Titanium plates have a limited role in paediatric orbital fractures and are deemed not suitable for use in growing children, owing to their rigid, unchanging dimensions, which predisposes to craniofacial growth restriction [15,16]. If used, titanium requires a secondary surgery for implant removal in the developing child [22]. Consideration may be given to older adolescent patients past the peak growth periods and in large orbital fractures [6,25]. Furthermore, there is a risk of plate migration, extrusion, fibrosis and orbital adherence syndrome with restriction of movement of extraocular eye muscles [15,21,22,86,87,89]. Novel materials such as combination (PPE and titanium) or composite materials (unsintered hydroxyapatite particles and PLLA) comprised a small proportion of materials. The current interpretations and generalisability of findings remain significantly limited by sample sizes and the low quality of studies. Their utility in paediatric orbital reconstruction remains a future avenue of investigation and research.

### 4.5. Clinical Relevance and Implications

Despite the methodological constraints of this systematic review, its findings may provide relevant information regarding material selection for paediatric orbital reconstruction. Autologous bone grafting remains widely utilised, owing to its biocompatibility [10,21,22,86] and low observed rates of late postoperative diplopia (0%) in the included studies. However, the morbidity associated with a second operative site must also be accounted for during clinical decision making. Furthermore, the unpredictable rate of resorption of autologous bone, may contribute to the observed incidence of enophthalmos (8%) [25]. Thus, autologous grafts may be more appropriate for smaller defects where the structural demands are limited. In contrast, porous polyethylene (including MedPor^®^) appears to be a good choice for larger defects, with lower reported enophthalmos rates (0%) observed in this review. This could be potentially attributable to its strength as well as its ability to provide support to the orbital contents, thus maintaining an appropriate orbital volume [21,25,91]. However, careful patient selection is required for porous polyethylene due to the higher associated late postoperative diplopia rates (6%). In younger patients (<7 years), where orbital growth is more pronounced, a slower-resorbing PLLA/PLA copolymer may be more appropriate to accommodate for the changes in dimensions over time [22]. Alternative and emerging materials such as xenografts, allografts, composites and combinations also remain available [20]. Robust evidence demonstrating the efficacy, longevity and safety of these materials has not yet been established. Optimal material selection requires individualisation, with the decision dependent on a balance of patient factors such as skeletal maturity, surgeon proficiency with specific implant systems, and the anatomical or structural demands of the fracture pattern.

### 4.6. Limitations

Despite this review offering a detailed analysis of material subtypes, providing granularity often absent in prior reviews, significant limitations are present. Given the paucity of literature pertaining to orbital reconstruction materials in the paediatric population, the available evidence is dominated by low-quality observational studies of a retrospective nature encompassing small cohorts, supplemented by case series and case reports, thus introducing a substantial risk of bias. Furthermore, there was considerable heterogeneity across studies, with a lack of standardisation in outcome reporting. Variable reporting of patient demographics, inconsistent documentation of follow-up, and a lack of detail regarding potential confounding factors such as fracture characteristics (size, complexity), surgical timing and operative techniques was also noted. This heterogeneity, in addition to the aforementioned low-quality of studies, significantly reduces the certainty of any comparative findings of the review. Furthermore, numbers from low-volume studies, case series and case reports may result in the zero-inflated modelling of outcomes, exaggerating apparent efficacy or safety. In the absence of high-quality, standardised, randomised controlled trials, the certainty and general applicability of findings—as well as causal inferences—cannot be firmly established. Assertions of comparative material superiority must not be made. Nonetheless, the notably low rates of severe complications such as vision loss, infection, implant removal, and reoperation across materials are reassuring, supporting the ongoing safe use of contemporary materials by experienced surgeons.

Further research conducted should aim to obtain high-quality primary research with prospective randomised multicentred controlled studies employing standardised outcome measures to elucidate material-specific advantages and disadvantages. Longer follow-up periods may also better quantify implant complications associated with growth-related changes in the paediatric craniofacial skeleton.

## 5. Conclusions

In conclusion, this systematic review evaluates existing low-quality literature surrounding the clinical outcomes of paediatric orbital fracture reconstructed with differing implant and graft materials. The included studies were predominantly observational in nature with significant methodological limitations. Autogenous bone grafts remain a common choice of reconstructive material due to their biocompatibility, integrative ability with lower observed late postoperative diplopia and restriction of EOM rates. Alloplastic and polymeric materials are also frequently utilised, and, together with autologous grafts, they comprise of the majority of material types used in paediatric orbital reconstruction. These material categories seem to exhibit heterogeneity in clinical outcomes, necessitating careful and individualised case selection. The review also highlights a sizable knowledge gap and the need for future high-quality primary studies to inform safe, evidence-based and individualised material selection in paediatric orbital fracture reconstruction.

## Figures and Tables

**Table 1:** Material categories and subtypes.

Material Category	Subtype/Example	*n* (%) (of Material)	% (of All Implants)
Polymers	Resorbable (PLLA/PLA/PGA combinations)	90/169 (53%)	16.0%
	Nylon foil	63/169 (37%)	11.2%
	Other (polydioxanone, polypropylene, polyester urethane etc.)	16/169 (9.5%)	2.8%
Alloplasts	Porous polyethylene (incl. Medpor)	120/167 (71.9%)	21.3%
	Medpor (as subgroup)	58/167 (34.7%)	10.3%
	Polytetrafluorethylene	45/167 (26.9%)	8.0%
	Other (including silicone)	2/167 (1.2%)	0.4%
Autologous	Bone graft (unspecified)	132/166 (79.5%)	23.4%
	Calvarial graft	16/166 (9.6%)	2.8%
	Iliac bone graft	2/166 (1.2%)	0.4%
	Other bone (lamina papyracea, rib)	6/166 (0.6%)	1.1%
	Cartilage (aurical/nasoseptal)	10/166 (6.0%)	1.8%
Xenograft	Lyoplant/Permacol (bovine collagen derivatives)	20/25 (80%)	3.6%
	Other (gelatin film, heterologous bone graft)	5/25 (20%)	0.9%
Allograft	Lyodura (lyophilised dura mater)	15/21 (71.4%)	2.7%
	Other (irradiated fascia lata, cadaveric cranial bone graft)	6/21 (28.6%)	1.1%
Metal	Titanium	6/6 (100%)	1.1%
Composite	Unsintered Hydroxyapatite & PLLA	5/5 (100%)	0.9%
Combination	PPE & Titanium	3/4 (75%)	0.7%
	Other (periosteum-polymer combination)	1/4 (25%)	0.2%

**Table 2:** Pairwise comparison (Fisher’s Exact Test) between materials, by outcome.

Outcome	Group Comparison	*p*-Value	Favoured Material (If Significant) and Comments
Late Diplopia			
	Autologous vs. Alloplast	0.002	Autologous
	Autologous vs. Polymer	<0.001	Autologous
	Porous Polyethylene vs. Bone Graft	<0.001	Bone Graft (Autologous)
	PLLA/PLA/PGA vs. Bone Graft	<0.01	Bone Graft (Autologous)
	Alloplast vs. Polymer	0.83	No significant difference
Enophthalmos			
	Alloplast vs. Autologous	<0.001	Alloplast
	Alloplast vs. Polymer	0.03	Alloplast
	Polymer vs. Autologous	0.07	No significant difference
EOM Restriction			
	Alloplast vs. Autologous	0.01	Autologous
	Polymer vs. Alloplast	0.06	No significant difference
	Polymer vs. Autologous	1.00	No significant difference

## Data Availability

Dataset available on request from the authors.

## Supplementary Materials

The following supporting information can be downloaded at: https://www.mdpi.com/article/10.3390/cmtr19010012/s1, Table S1: Complications by Material Type and Clinical Outcome; Table S2: Newcastle-Ottawa Scale (NOS) Risk of Bias Assessment for Observational Studies; Table S3: JBI Critical Appraisal for Case Series; Table S4: JBI Critical Appraisal for Case Reports.

## Abbreviations

The following abbreviations are used in this manuscript:

EOM	Extraocular eye muscles
PRISMA	Preferred Reporting Items for Systematic Review and Meta-analyses
RCT	Randomised control trial
RTT	Return to theatre
NOS	Newcastle–Ottawa Scale
JBI	Joanna Briggs Institute
PLA	Polylactic acid
PLLA	Poly-L-lactide acid
PGA	Polyglycolic acid
PPE	Porous polyethylene
