# Supplementary material for: Comparison of Reconstructive Materials in Paediatric Orbital Fractures: A Systematic Review
Source: Craniomaxillofac Trauma Reconstr. 2026 Feb 23;19(1):12. doi: 10.3390/cmtr19010012 (PMC13024892; doi:10.3390/cmtr19010012)
Supplement: Supplementary file 1 [file cmtr-19-00012-s001.zip › cmtr-4077239-supplementary.pdf]

**Supplementary Materials**

**Table S1. Complications by Material Type and Clinical Outcome .**

| Material | Allograft, *n* = 21 | 95% CI | Alloplast, *n* = 167 | 95% CI | Autologous, *n* = 166 | 95% CI | Combination, *n* = 4 | 95% CI |
|---|---|---|---|---|---|---|---|---|
| **Diplopia, Early (<2 weeks), *n* (%)** | 0 (0.0%) | 0 – 16.1 | 19 (11.4%) | 7 – 17.2 | 0 (0.0%) | 0 – 2.2 | 0 (0.0%) | 0 – 60.2 |
| **Diplopia, Late, *n* (%)** | 6 (28.6%) | 11.3 – 52.2 | 10 (6.0%) | 2.9 – 10.7 | 0 (0.0%) | 0 – 2.2 | 0 (0.0%) | 0 – 60.2 |
| **Hypoglobus, *n* (%)** | 0 (0.0%) | 0 – 16.1 | 0 (0.0%) | 0 – 2.2 | 3 (1.8%) | 0.4 – 5.2 | 0 (0.0%) | 0 – 60.2 |
| **Enophthalmos, *n* (%)** | 0 (0.0%) | 0 – 16.1 | 0 (0.0%) | 0.0 – 2.2 | 14 (8.4%) | 4.7 – 13.7 | 0 (0.0%) | 0 – 60.2 |
| **Eye movement restriction, *n* (%)** | 0 (0.0%) | 0 – 16.1 | 7 (4.2%) | 1.7 – 8.4 | 0 (0.0%) | 0 – 2.2 | 0 (0.0%) | 0 – 60.2 |
| **V2 paraesthesia, *n* (%)** | 0 (0.0%) | 0 – 16.1 | 0 (0.0%) | 0 – 2.2 | 0 (0.0%) | 0 – 2.2 | 1 (25.0%) | 0.6 – 80.6 |
| **Removal of material, *n* (%)** | 0 (0.0%) | 0 – 16.1 | 1 (0.6%) | 0 – 3.3 | 1 (0.6%) | 0 – 3.3 | 0 (0.0%) | 0 – 60.2 |

| **Material** | **Allograft, *n* = 21** | 95% CI | **Alloplast, *n* = 167** | 95% CI | **Autologous, *n* = 166** | 95% CI | **Combination, *n* = 4** | 95% CI |
|---|---|---|---|---|---|---|---|---|
| **Vision loss, *n* (%)** | 0 (0.0%) | 0 – 16.1 | 1 (0.6%) | 0 – 3.3 | 0 (0.0%) | 0 – 2.2 | 0 (0.0%) | 0 – 60.2 |
| **Return to theatre, *n* (%)** | 0 (0.0%) | 0 – 16.1 | 3 (1.8%) | 0.4 – 5.2 | 5 (3.0%) | 1 – 6.9 | 0 (0.0%) | 0 – 60.2 |
| **Infection, *n* (%)** | 0 (0.0%) | 0 – 16.1 | 2 (1.2%) | 0.1 – 4.3 | 2 (1.2%) | 0.1 – 4.3 | 0 (0.0%) | 0 – 60.2 |
| **Complication - other, *n* (%)** | 0 (0.0%) | 0 – 16.1 | 3 (1.8%) | 0.4 – 5.2 | 4 (2.4%) | 0.7 – 6.1 | 0 (0.0%) | 0 – 60.2 |

| **Material** | **Composites, *n* = 5** | 95% CI | **Metal, *n* = 6** | 95% CI | **Polymers, *n* = 169** | 95% CI | **Xenograft, *n* = 25** | 95% CI |
|---|---|---|---|---|---|---|---|---|
| **Diplopia, Early (<2 weeks), *n* (%)** | 0 (0.0%) | 0 – 52.2 | 0 (0.0%) | 0 – 45.9 | 22 (13.0%) | 8.3 – 19 | 2 (8.0%) | 1 – 26 |
| **Diplopia, Late, *n* (%)** | 0 (0.0%) | 0 – 52.2 | 2 (33.3%) | 4.3 – 77.7 | 12 (7.1%) | 3.7 – 12.1 | 6 (24.0%) | 9.4 – 45.1 |
| **Hypoglobus, *n* (%)** | 0 (0.0%) | 0 – 52.2 | 0 (0.0%) | 0 – 45.9 | 0 (0.0%) | 0 – 2.2 | 0 (0.0%) | 0 – 13.7 |

| | | | | | | | | |
|---|---|---|---|---|---|---|---|---|
| **Enophthalmos, *n* (%)** | 0 (0.0%) | 0 – 52.2 | 0 (0.0%) | 0 – 45.9 | 6 (3.6%) | 1.3 – 7.6 | 0 (0.0%) | 0 – 13.7 |
| **Eye movement restriction, *n* (%)** | 0 (0.0%) | 0 – 52.2 | 0 (0.0%) | 0 – 45.9 | 1 (0.6%) | 0 – 3.3 | 0 (0.0%) | 0 – 13.7 |
| **V2 paraesthesia, *n* (%)** | 0 (0.0%) | 0 – 52.2 | 0 (0.0%) | 0 – 45.9 | 2 (1.2%) | 0.1 – 4.2 | 0 (0.0%) | 0 – 13.7 |
| **Removal of material, *n* (%)** | 0 (0.0%) | 0 – 52.2 | 0 (0.0%) | 0 – 45.9 | 4 (2.4%) | 0.6 – 5.9 | 1 (4.0%) | 0.1 – 20.4 |
| **Vision loss, *n* (%)** | 0 (0.0%) | 0 – 52.2 | 0 (0.0%) | 0 – 45.9 | 0 (0.0%) | 0 – 2.2 | 0 (0.0%) | 0 – 13.7 |
| **Return to theatre, *n* (%)** | 0 (0.0%) | 0 – 52.2 | 0 (0.0%) | 0 – 45.9 | 4 (2.4%) | 0.6 – 5.9 | 1 (4.0%) | 0.1 – 20.4 |
| **Infection, *n* (%)** | 0 (0.0%) | 0 – 52.2 | 0 (0.0%) | 0 – 45.9 | 1 (0.6%) | 0 – 3.3 | 0 (0.0%) | 0 – 13.7 |
| **Complication - other, *n* (%)** | 0 (0.0%) | 0 – 52.2 | 0 (0.0%) | 0 – 45.9 | 5 (3.0%) | 1.0 – 6.8 | 1 (4.0%) | 0.1 – 20.4 |

**Table S2.** Newcastle-Ottawa Scale (NOS) Risk of Bias Assessment for Observational Studies.

| Study ID | Study Design | Selection (Max 4) | Comparability (Max 2) | Outcome (Max 3) | Total Score | Quality Classification |
|---|---|---|---|---|---|---|
| Jordan 1992 [56] | Retrospective cohort | 2 | 0 | 0 | **2** | Poor |
| Bedrossian 1993 [41] | Retrospective cohort | 2 | 0 | 2 | **4** | Poor |
| Mauriello 1994 [68] | Retrospective cohort | 2 | 0 | 1 | **3** | Poor |
| McVicar 1995 [70] | Retrospective cohort | 2 | 0 | 3 | **5** | Poor |
| Kumar 1997 [62] | Retrospective cohort | 2 | 0 | 0 | **2** | Poor |
| Lai 1998 [63] | Retrospective cohort | 3 | 0 | 3 | **6** | Poor |
| Choi 1999 [46] | Prospective cohort | 2 | 0 | 3 | **5** | Poor |
| Baek 2003 [39] | Retrospective cohort | 3 | 0 | 1 | **4** | Poor |
| Yavuzer 2004 [84] | Retrospective cohort | 2 | 0 | 1 | **3** | Poor |
| Ozyazgan 2006 [75] | Retrospective cohort | 2 | 0 | 3 | **5** | Poor |
| Lin 2007 [65] | Retrospective cohort | 2 | 0 | 3 | **5** | Poor |
| Theologie-Lygidakis 2007 [11] | Retrospective cohort | 4 | 0 | 3 | **7** | Poor |
| Tuncer 2007 [82] | Retrospective cohort | 2 | 0 | 3 | **5** | Poor |
| Asamura 2010 [37] | Retrospective cohort | 3 | 0 | 2 | **5** | Poor |

| Study ID | Study Design | Selection (Max 4) | Comparability (Max 2) | Outcome (Max 3) | Total Score | Quality Classification |
|---|---|---|---|---|---|---|
| Cheong 2010 [44] | Retrospective cohort | 3 | 0 | 2 | **5** | Poor |
| Gerbino 2010 [50] | Retrospective cohort | 4 | 0 | 3 | **7** | Poor |
| Iatrou 2010 [54] | Retrospective cohort | 2 | 0 | 1 | **3** | Poor |
| Kim 2010 [59] | Retrospective cohort | 3 | 0 | 1 | **4** | Poor |
| Nowinski 2010 [74] | Retrospective cohort | 4 | 0 | 3 | **7** | Poor |
| Ng 2012 [72] | Retrospective cohort | 4 | 0 | 3 | **7** | Poor |
| Zunz 2012 [86] | Retrospective cohort | 2 | 0 | 1 | **3** | Poor |
| Hink 2014 [53] | Retrospective cohort | 3 | 0 | 1 | **4** | Poor |
| Lin 2014 [66] | Retrospective cohort | 2 | 0 | 1 | **3** | Poor |
| Timoney 2014 [81] | Retrospective cohort | 3 | 0 | 1 | **4** | Poor |
| Kanno 2017 [57] | Retrospective cohort | 2 | 0 | 3 | **5** | Poor |
| Abumanhal 2019 [34] | Retrospective cohort | 3 | 0 | 3 | **6** | Poor |
| Dong 2020 [48] | Retrospective cohort | 2 | 0 | 3 | **5** | Poor |

| Study ID | Study Design | Selection (Max 4) | Comparability (Max 2) | Outcome (Max 3) | Total Score | Quality Classification |
|---|---|---|---|---|---|---|
| Chai 2021 [43] | Retrospective cohort | 3 | 0 | 3 | **6** | Poor |
| Koryczan 2021 [60] | Retrospective cohort | 3 | 0 | 0 | **3** | Poor |
| Zhao 2022 [85] | Retrospective cohort | 3 | 0 | 2 | **5** | Poor |
| Cena 2025 [42] | Retrospective cohort | 4 | 0 | 3 | **7** | Poor |

**Table S3.** JBI Critical Appraisal for Case Series.

| **Study ID** | **Inclusion Criteria** | **Condition Measured Reliably** | **Valid Identification Method** | **Consecutive Inclusion** | **Complete Inclusion** | **Reporting of Demographics** | **Clear Clinical Information** | **Outcomes Reported Clearly** | **Appropriate Statistical Analysis** |
|---|---|---|---|---|---|---|---|---|---|
| Kraus 2002 [61] | Yes | Yes | Yes | Unclear | Unclear | Yes | No | Yes | No |
| Taban 2009 [80] | Yes | Yes | Yes | Yes | Yes | Yes | Yes | Yes | Yes |
| Segna 2016 [76] | No | Yes | Yes | Unclear | Unclear | Yes | Unclear | Yes | No |
| Niklinska 2021 [73] | Yes | Yes | Yes | Unclear | Yes | Yes | Yes | Yes | Yes |
| Basnet 2024 [40] | Yes | Yes | Yes | Unclear | Unclear | Yes | Yes | Yes | No |

**Table S4.** JBI Critical Appraisal for Case Reports.

| Study ID | Demographic Characteristics | Patient History Described | Clinical Condition Described | Diagnostic Tests Described | Intervention Described | Post-Intervention Condition Described | Adverse Events Described | Takeaway Lessons Described |
|---|---|---|---|---|---|---|---|---|
| Cheung 2004 [45] | Yes | Yes | Yes | Yes | No | Yes | Yes | Yes |
| Babar 2009 [38] | Yes | Yes | Yes | Yes | Yes | Yes | No | Yes |
| McInnes 2010 [69] | Yes | No | Yes | Yes | Unclear | Yes | No | Yes |
| Lee 2014 [64] | Yes | No | Yes | Yes | Yes | Yes | No | Yes |
| Maloney 2014 [67] | Yes | No | Yes | Yes | Yes | Yes | No | Yes |
| He 2017 [52] | Yes | Yes | Yes | Yes | Unclear | Yes | No | Yes |
| Dunphy 2019 [49] | Yes | Yes | Yes | Yes | No | Yes | No | Yes |
| Shaughness 2019 | Yes | Yes | Yes | Yes | Unclear | Yes | Yes | Yes |
| Valente 2019 [83] | Yes | Unclear | Yes | Yes | Yes | Yes | No | Yes |
| Sobol 2020 [79] | Unclear | No | Yes | Yes | Yes | Yes | No | Yes |
| Akiki 2021 [35] | Yes | Yes | Yes | Yes | Yes | Yes | No | Unclear |
| Kerdoud 2021 [58] | Yes | Yes | Yes | Yes | Yes | Yes | Yes | Yes |

| Study ID | Demographic Characteristics | Patient History Described | Clinical Condition Described | Diagnostic Tests Described | Intervention Described | Post-Intervention Condition Described | Adverse Events Described | Takeaway Lessons Described |
|---|---|---|---|---|---|---|---|---|
| Amarath-Madav 2022 [36] | Yes | Yes | Yes | Yes | Yes | Yes | No | Yes |
| Nadershah 2022 [71] | Yes | No | Yes | Yes | Yes | Yes | No | Yes |
| Dehghanpour Baruoj 2023 [47] | Yes | Yes | Yes | Yes | Yes | Yes | No | Yes |
| Sharma 2023 [77] | Yes | No | Yes | Yes | Yes | Yes | No | Yes |
| Imagawa 2024 [55] | Yes | Yes | Yes | Yes | Yes | Yes | No | Yes |
| Hakkou 2025 [51] | Yes | Yes | Yes | Yes | No | Yes | No | Yes |
